# Knowledge and practice of adolescent females about menstruation and menstruation hygiene visiting a public healthcare institute of Quetta, Pakistan

**DOI:** 10.1186/s12905-019-0874-3

**Published:** 2020-01-06

**Authors:** Judy Michael, Qaiser Iqbal, Sajjad Haider, Adnan Khalid, Naheed Haque, Rabia Ishaq, Fahad Saleem, Mohamed Azmi Hassali, Mohammad Bashaar

**Affiliations:** 1 Faculty of Pharmacy & Health Sciences, University of Balochistan, Quetta, Pakistan; 2 Combined Military Hospital, Quetta, Pakistan; 3 School of Pharmaceutical Sciences, Universiti Sains Malaysia, Penang, Malaysia; 4 SMART Afghan International Trainings & Consultancy, Kabul, Afghanistan

**Keywords:** Knowledge, Practice, Menstruation, Menstruation hygiene, Adolescent

## Abstract

**Background:**

The current study is aimed to assess menstruation-related knowledge and practices of adolescent females visiting a public health care institute of Quetta city, Pakistan.

**Methods:**

A questionnaire-based cross-sectional survey was conducted. Nine hundred and twenty three female adolescents attending general out-patient departments of Mohtarma Shaheed Benazir Bhutto Hospital Quetta, Balochistan, was approached for data collection. Based on the objectives of the study, descriptive analysis was conducted and SPSS v. 21.0 was used for the data analysis.

**Results:**

Demographic characteristics revealed that the mean age of the respondents was 15 years. Mothers’ (67%) were the main source of menstruation-related information. Majority (77.7%) of our respondents never had a class or session regarding menstruation-related education in their schools. About (44%) knew that menstruation is a physiological phenomenon while 60.2% knew that menstrual blood comes from the vagina. Nearly 40% of our study respondents missed their schools because of menarche. The use of absorbent material was frequent (90%) among the adolescent females and (68.7%) used commercially available sanitary napkins/pads. Although majority of the respondents (58.2%) were not taking baths during menstruation, 80.5% do cleaned their genitalia with water during menstruation.

**Conclusion:**

Female adolescents of our study had certain misconception regarding menstruation because of poor access to health-related education. Education can be provided at healthcare facilities, residential area as well as religious centers. Adolescent reproductive health should be included in the school curriculum; this will influence general reproductive health of females.

## Background

Menstruation is defined as “*periodic discharge of blood from the uterus occurring more or less at regular monthly intervals throughout the active reproductive life of a female*” [1]. While menarche is a natural event in girls’ life, it is also the beginning of physical and social evolution [2]. Even though the occurrence of regular menstruation is an evidence of a female’ excellent reproductive health, the very menstruation is misconstrued as detrimental and adulterated in many societies [3–5]. Additionally, for certain societies it is prohibited to talk about menstruation and the related issues [6]. Therefore, a natural biological process that dignifies women and girls becomes a subject of shame and annoyance. Such social taboos and myths increase the vulnerability of post pubescent females to unhygienic practices that lead to multiple infections including reproductive and urinary tract [7]. What’s more, the societal restriction and scarcity of menstrual-related information results in social dishonor that affect girls’ physical and emotional state [6]. This ends in loneliness, school absenteeism, and limited academic, sports and daily activities, hence partially or completely isolating the girl from the society [8].

Shifting our concerns to health-related issues of menstruating girls and women, menstrual sanitation and performance are influenced by multiple factors, however; knowledge about menstruation plays a key role in attaining proper hygiene [9, 10]. High-quality menstrual hygienic knowledge and practices are essential throughout menstruation time as it improve the self-assurance of females in several ways [11, 12]. Nevertheless, research and dissemination of accurate information about menstruation has been neglected especially in the developing world for a long time [13]. Chandra-Mouli and Patel in their review reported that girls in majority of the developing world enter puberty with knowledge gaps and mistaken beliefs about menstruation. The authors argued that this lack of knowledge is because adults around them are ill-informed and uncomfortable discussing issues related to sexuality, reproduction and menstruation [14]. These findings are further supported by various studies reported from South Asia where girls had poor knowledge of menstruation and thus do not fully understood the physical process of menstruation [15]. Inline to what is reported; Coast et al. in their scoping review claimed about inadequate knowledge about menstruation and low levels of pre-menarche knowledge among adolescent girls, hence were under-prepared for puberty and menstruation [16].

In addition to poor knowledge towards menstruation, females in low- and middle income countries are normally deprived in making use of the available resources, especially when it comes to menstruation-related hygiene issues. Within this context, no formal guidance from the schools, lack of cultural acceptance of alternative menstrual products, limited economic resources and inadequate water and sanitation facilities are reported as major reasons [17]. Moreover, inadequate WASH (water, sanitation and hygiene) facilities, particularly in schools and other public places are rated as a major obstacle. Collectively, a growing body of evidence shows that girls’ inability to manage their menstrual hygiene results in school absenteeism, poor academic performance and episodes of anxiety and depression during or near the menstrual period which in turn has severe societal, economic, healthcare costs [18]. Therefore, evaluating menstrual knowledge and hygiene performance of female adolescents and understanding the knowledge gap is crucial to strengthen sanitized practices during the menstruation cycle [19].

As reported from other developing countries, menstruation is also linked with several misconceptions and poor practices in Pakistan and menstrual practices are clouded by taboos and socio-cultural restrictions [20]. The social stigma attached to menstruation causes many girls to carryout unsafe hygiene practices which in return places a burden on the social, economical and the healthcare system [20]. It is important to highlight that the mentioned evidence reported from Pakistan is from metropolitan cities and there is scarcity of information from remote areas such as from Quetta city, Balochistan [21]. In addition, being a conservative and tribal area, we believe that the social taboos and myths are more prominent as compared to other parts of the country. Keeping the paucity of data from the current study location, our study was aimed to assess the menstruation-related knowledge and practices of adolescent females visiting a public health care institute of Quetta city, Pakistan. Likewise, results of the current study will try to identify menstruation-related issues that are not previously reported in literature.

## Methods

### Study design and settings

A questionnaire-based cross-sectional survey was conducted. Data were collected from patients attending the general outpatient department (OPD) of Mohtarma Shaheed Benazir Bhutto Hospital Quetta (MSBBH), Balochistan. Quetta is the provincial capital and largest city of Balochistan province, Pakistan. Located in northern Balochistan near the Pakistan-Afghanistan border, Quetta is a trade and communication centre between the two countries. This hospital is among the four biggest government hospitals of Quetta city and provides major healthcare facilities to the general population [22].

### Study population, sampling and inclusion criteria

The target population was female adolescents. According to the World Health Organization (WHO) criterion, teenage begins with the onset of puberty and ends when individuality and conduct is maintained. This period expands between 10 and 19 years of age [23]. Proportionate based sampling with a double design effect method was employed for sample size calculation and 940 respondents were targeted for data collection [24].

### Data collection tool

In addition to the demographic information, a structured questionnaire was constructed (Additional file 1) based on extensive literature review [25–27]. The questionnaire was translated in Urdu (Additional file 2, National language and lingua franca of Pakistan) by a linguistic expert; the questionnaire was then translated back into English by another expert to avoid any discrepancy in the two versions. Face and content validity was established by physicians, their opinion was taken into consideration before the pilot study. The questionnaire was subjected to a pilot analysis with 30 participants. The questionnaire was declared reliable with an acceptable alpha value of 0.8. Data from the pilot study was not included in the field study.

### Data analysis

SPSS version 21 was used for the analysis of the data. Based on the objectives of the study, descriptive analysis was conducted.

## Results

Data were available from 923 respondents with a response rate of 98.19%. Table 1 demonstrates the demographic characteristics of the study respondents. The mean age of the respondents was 15 years. Majority of the respondents (241, 26.1%) had the age of 17 years and 51% had an intermediate level of education. Menarche was reported at the age range of 12–14 years among 73 % of the respondents.

**Table 1:** Characteristics of the study respondents

Characteristics	*N* = 923	%
Age in Years		
12	131	14.2
13	26	2.8
14	110	11.9
15	180	19.5
16	109	11.8
17	241	26.1
18	126	13.7
Educational Level		
Middle (8th standard)	128	13.8
Matriculation (10th standard)	318	34.4
Intermediate (12th standard)	477	51.6
Religion		
Islam	832	90.1
Christianity	84	9.1
Hinduism	7	0.8
Father’ Education		
Illiterate	43	4.7
Primary	485	52.5
Middle	45	4.9
Matriculation	59	6.4
Intermediate	291	31.5
Mother’s Education		
Illiterate	115	12.5
Primary	344	37.3
Middle	100	10.8
Matriculation	84	9.1
Intermediate	280	30.3
Father’s Occupation		
Business	223	24.2
Government employee	486	52.7
Non-Government employee	78	8.5
Unemployed	11	1.2
Retired	33	3.6
Others	92	10.0
Mother’s Occupation		
Housewife	712	77.13
Government employee	155	16.8
Non-Government employee	28	3.0
Unemployed	10	1.1
Retired	8	.9
Others	10	1.1
Age at menarche		
Before 11 Years	77	8.3
12–14 Years	675	73.1
15–16 Years	171	18.5

Table 2 presents the sources of information regarding menstruation. Mothers were the main source of information to the adolescent girls (618, 67%) before the menstrual period, and 366 (39.7%) of the respondents had information that menstruation bring physical changes. On the contrary, a large number of study respondents (717, 77.7%) did not attend a class or session about menstruation at their schools.

**Table 2:** Source of information regarding menstruation

Questions	*N* = 923	%
Source of knowledge of menstruation before the beginning of the menstrual period.		
Mother	618	67.0
Elder sister	172	18.6
Aunt	29	3.1
Friend	76	8.2
Media	7	0.8
Others	21	2.3
Information about menstruation before the beginning of the menstrual period.		
Physical changes	366	39.7
Social Religious restrictions	59	6.4
Bathing processes	55	6.0
Use of the material for absorption	99	10.7
The blood is dirty	93	10.1
The blood comes from the vagina	80	8.7
None	171	18.5
Before the onset of menstruation, have you had any class session related to menstruation at your school?		
Yes	206	22.3
No	717	77.7

Table 3 presents knowledge of the study respondents regarding menstruation. A large number of respondents (407, 44.1%) knew that menstruation is a physiological process and is caused by hormones (642, 69.6%) while, (207, 22.4%) had no idea about menstruation and its cause (140, 15.2%). Five hundred and fifty six girls knew that menstrual blood discharges from vagina, and (237, 25.7%) thought that at the age of 13, girls usually get their first period. Almost 90 % of the respondents knew how to use sanitary pads, and (560, 60.7%) thought more nutritious diet is required during periods. Two hundred and fifty one (27.2%) had menstruation flow of seven days and (740, 80.2%) thought menstrual blood is unhygienic.

**Table 3:** Knowledge of respondents regarding menstruation

Questions	*N* = 923	%
What is menstruation?		
Physiological Process	407	44.1
Disease	28	3.0
Curse of God	250	27.1
Others	31	3.4
Don’t know	207	22.4
What is the cause of menstruation?		
Hormones	642	69.6
Curse of God	114	12.4
Disease	19	2.1
Others	8	0.9
Don’t know	140	15.2
From which organ does the menstrual blood discharge from?		
Uterus	161	17.4
Vagina	556	60.2
Bladder	35	3.8
Abdomen	14	1.5
Others	8	0.9
Don’t know	149	16.1
At what age do you think girls usually get their first period?		
10	20	2.2
11	87	9.4
12	233	25.2
13	237	25.7
14	222	24.1
15	70	7.6
16	43	4.7
17	6	0.7
18	5	0.5
Do you know how to use a sanitary pad?		
Yes	838	90.8
No	85	9.2
Do you know that girls should take more nutritious diet during their periods?		
Yes	560	60.7
No	118	12.8
Don’t know	245	26.5
What is the average duration of your menstruation flow?		
4	193	20.9
5	192	20.8
6	201	21.8
7	251	27.2
8	71	7.7
9	8	0.9
10	7	0.8
Do you think the menstrual blood is unhygienic?		
Yes	740	80.2
No	183	19.8

Table 4 indicates problems faced by respondents during menstruation. In response to problems associated with menstruation (286, 31%) reported back pain, (266, 28.8%) reported abdominal pain while, (230, 24.9%) reported general weakness. Moreover, 61.5% didn’t miss school due to menarche while 53.2% sometimes missed other activities during menstruation. Almost 50% avoided certain foods and 70.1% felt there is foul smell during menstruation.

**Table 4:** Problems faced by respondents during menstruation

Questions	*N* = 923	%
Do you have any problems associated with menstruation?		
Headache	59	6.4
Vomiting	44	4.8
Weakness	230	24.9
Anorexia	8	0.9
Abdominal pain	266	28.8
Back pain	286	31.0
Others	30	3.3
Have you missed school because of menarche?		
Yes	355	38.5
No	568	61.5
Do you ever miss other activities (sports, games, social gatherings, etc.) during menstruation?		
Not at all	242	26.2
Sometimes	491	53.2
Rarely	85	9.2
Always	105	11.4
Do you avoid certain foods during menstruation?		
Yes	479	51.9
No	444	48.1
Do you think there is foul smell during menstruation?		
Yes	647	70.1
No	276	29.9

Table 5 reports the reaction of the respondents towards menstruation. In reaction to first menstruation (399, 43.2%) felt discomfort and (261, 28.3%) were scared. Six hundred and thirty four (68.7%) used commercially made sanitary napkins/pads and (632, 68.5%) didn’t take any medication for the associated problems. To ease the discomfort, rest was taken by (626, 67.8%) respondents while, (537, 58.2%) didn’t take bath during the menstruation period. Majority (57.6%) used water to clean their genitalia during menstruation.

**Table 5:** Reaction of the respondents towards menstruation

Questions	*N* = 923	%
What was the reaction to your first menstruation?		
Happy	59	6.4
Scared	261	28.3
Discomfort	399	43.2
Emotional disturbance	194	21.0
Others	10	1.1
What are your eating habits during menstruation?		
Eat less	356	38.6
Eat more	141	15.3
Eat same amount of food	426	46.2
Do you use absorbent material during period?		
Yes	815	88.3
No	108	11.7
What absorbent material do you use during menstruation? (You may choose more than one option)		
Commercially made sanitary napkin/pad	634	68.7
Homemade pads	150	16.25
Cotton wool	107	11.6
Nothing	8	0.9
Other	24	2.6
Do you take any medications for problems associated with menstruation?		
Yes	291	31.5
No	632	68.5
What other remedies do you use to ease the discomfort of menstruation?		
Rest	626	67.8
Oil massage	32	3.5
Turmeric milk	102	11.1
Hot bottle packs	113	12.2
Others	50	5.4
When do you prefer to take bath during menstrual period?		
Daily	72	7.8
First day	56	6.1
Second day	186	20.2
Not during the period	537	58.2
Others	72	7.8
Do you clean your genitalia during menstruation?		
Yes	743	80.5
No	180	19.5
If yes, mostly with what?		
Water and soap	234	25.4
Only with water	532	57.6
Tissue paper	102	11.1
Towel	6	0.7
Others	49	5.3

Table 6 shows the handling of the used material by the respondents. Used material was discarded by 65% of the respondents. The dustbins were used to dispose of the material by 75% of the respondents. Forty five percent of the respondents changed the adsorbent pad twice a day.

**Table 6:** Handling of the used material by the study respondents

Questions	*N* = 923	%
How do you handle the used material?		
Discard it	607	65.8
Wash and discard it	226	24.5
Wash and reuse	90	9.8
Where do you dispose off your used materials?		
Dustbin	692	75.0
Drains	39	4.2
Toilets	61	6.6
Burn them	107	11.6
Others	24	2.6
How many times a day do you change the absorbent cloth/pad		
Once	127	13.8
Twice	424	45.9
Thrice	245	26.5
More than 3 times	127	13.8

## Discussion

Because of the scarcity of literature reported from Quetta city, the current study was aimed to assess the menstruation-related knowledge and practices of adolescent females. Results of our study revealed mothers as major source of menstruation-related information for the respondents followed by their sisters Table 2). Our results are in line to what is reported from India, Nepal and South Africa [28, 29]. Even though the evidence declared menstruation as an uncomfortable topic especially for preteen girls to discuss; mothers and sisters do play a significant role in conveying information about physiological changes and also about social, emotional and cultural issues. However, the reliability of the menstruation-related information provided by mothers and sisters is questionable as they are not necessarily well equipped to fill gaps in girls’ knowledge [14]. It is important to make sure that accurate and reliable information is transferred so that the menstruating girls are prepared to handle menstruation-related issues. Hennegan et al. in their response to the editorial did mention that majority of the menstruation-related findings and figures are not substantiated by published peer-reviewed studies and the original sources remain known [30]. Thus, assessment and correction of mothers’ and sisters’ menstruation-related knowledge is important and should be a matter of concern for healthcare professionals and local support groups.

Parallel to our study results, study respondents from Saudi Arabia and India considered menstruation a natural phenomena and a normal physical change [31, 32]. Importantly and as expected, 80% of our study participants had no prior classes or discussions about menstruation and hygiene at school. The finding is not surprising from a developing world as many girls receive no information or training from schools on how to manage menstruation-related issues [33]. Continuing with our arguments, Kaur et al. discussed that the education sector of developing countries avoids issues related to the menstruation and menstrual hygiene management by considering it a personal matter. Besides, teachers are also not ready to discuss menstruation and menstrual hygiene management with their students. Due to the unsupportive environment, girls remain unfamiliar with menstrual-related issues, and how to take care of personal hygiene [34].

In contrast to the findings of Khanna et al., where 97.5% of their participants didn’t know the source of bleeding, majority of the respondents, in the current study knew that vagina is the organ from where the menstrual blood flows [32]. In our study, 69.6% respondents knew that menstruation is caused by hormonal changes and adolescent girls had adequate knowledge of the duration of the menstrual cycle, similar to that found in Nigeria [35]. In continuation, Dasgupta and Sarkar in their study revealed that few adolescent females perceived menstrual blood as unhygienic, which is similar to the perception of our participants [9].

Almost 40% of our study respondents missed their school which is also supported by literature. The relationship between menstruation and school absenteeism is multi factorial. Even though, the present study was not able to identify such factors, we do hypothesize lack of facilities at schools and privacy as major factors. To support our hypothesis, Miiro and colleagues reported that menstruation was strongly associated with school attendance [36]. The United Nations Children’s Fund (UNICEF) estimated that about one in ten school-age African girl didn’t attend school during menstruation due to lack of cleanliness and separate toilet facilities for female students at schools [37]. Girls are frequently faced with difficulties in managing menstrual periods at school due to lack of adequate privacy and sanitary facilities [38]. As a result girls preferred to stay at home during their menstruation period. In addition to the school, our study respondents also missed out other activities, particularly religious ceremonials. In India, girls were restricted from cooking food and visiting temples during the menstruation period [6]. The influence of religion in the developing world is not to be underestimated and the same influence can be used to answer menstruation-related issues. Religious leaders of the developing world (Imams, Pandits, Monks and Priests) can be trained and used as a source of evidence based knowledge about menstruation and because of their influence on general public can prove to be a useful resource in handling issue related to menstruation.

Adolescent females of the current study were frightened and felt discomfort during their menarche which is supported by a study conducted in Mashhad, Iran [39]. Our findings are further strengthened as Hennegen et al. in their systematic review and qualitative meta-synthesis stated that menstruation is experienced with discomfort and fear [40]. Furthermore, a large number of our respondents reported usual eating habits during menstruation time. This is against to what is reported by Meyer-Rochow who explained food taboos are directly related to certain events including menstruation [41]. According to Khan (2000), adolescents and their mothers believed that eating foods considered to be too hot (dry fruits, liver, and eggs) or too cold (ice creams, yogurt, and green leafy vegetables) should be avoided [42]. A possible explanation of this difference is the negligible or poor existence of food taboos in the Pakistani society. Other then what is prohibited by the religion, food is considered as a source of vitality and is the reason of regular heating habits among the adolescent girls during their menstruation time.

Almost 70% of our respondents were using commercially available sanitary pads or napkins as an adsorbent material. This finding is against to another study conducted in Pakistan whereby an under usage of napkins due to lack of affordability was reported. The author also reported comfort of using homemade pads [43]. A possible explanation of this difference is the long gap between the two studies and since then much has evolved in terms of knowledge dissemination (especially the evolution of social media). Besides that, our respondents are urban residents while the participants of Khan’ study belonged to a rural settings. Nonetheless, the use and reuse of homemade napkins is frequently reported from low and middle income countries [17]. Additionally 80% of the current study respondents reported to use water and soap for cleaning their genitalia. On the contrary, a study done in Andhra Pradesh revealed that only 4.6% of the students used water and soap to clean their genitalia that again is related to economical and availability issues [35].

Most of the participants in the present study used commercially available pads and changed their sanitary pads on a daily basis. On average, our respondents used two pads in a day. This is against to the findings of a study conducted in Karachi, Pakistan where the authors claimed that girls in Karachi are either unaware of how to manage menstruation in a hygienic manner or they cannot afford to manage it with modern menstrual materials [20]. In other studies, although the use of pads was preferred over homemade rags and clothes, affordability was rates as a barrier [44, 45]. In certain cases there was no issue of affordability, the pads were thought to be uncomfortable as irritation or rashes were reported by the users. Hence, some female preferred new cloths and towels during menstruation, which were reused after being, washed [46]. Within this context, it is to remember that all those who menstruate need access to their choice of menstrual materials that are affordable, safe and comfortable. We have to remember that menstruation is a normal physiological process in females, but poor hygiene can bring serious health issues like urinary and reproductive tract infection. Therefore, females, especially adolescents, must be psychologically prepared about the physical changes that occur during this period. They should be well aware of the menstruation, menstrual hygiene, and menstrual cycle even before their menarche as well as how to maintain hygiene during the cycle.

## Conclusion

Concluding our study, female adolescents had certain misconceptions regarding menstruation because of poor access to health-related education. These misconceptions impact their practices to manage menstruation-related issues. Accurate and reliable knowledge can influence attitudes and practices over time, therefore individuals and organizations involved in women’ health have to intervene and improve awareness among adolescents regarding menstruation-related issues. Healthcare institute, schools, local societal settings and religious centers should be used to disseminate menstruation-related information among adolescents that will facilitate the transfer of quality menstruation-related practices to the next generation.

### Limitations

Because of the descriptive nature of study, we were unable to explore the relationship between menarche and school dropout. This should be investigated in another study in near future.

## Supplementary information

**Additional file 1.** Questionnaire used for data collection (English version).

**Additional file 2.** Questionnaire used for data collection [Urdu (National language of Pakistan) version].

## Data Availability

The datasets used and analyzed during the current study are available from the corresponding author at dr.mbashaar@gmail.com on request.

## Abbreviations

MSBBH: Mohtarma Shaheed Benazir Bhutto Hospital
OPD: Outpatient department
UNICEF: United Nations Children’s Fund
WASH: Water, Sanitation and hygiene
WHO: World Health Organization
