# Supplementary material for: Knowledge and practice of adolescent females about menstruation and menstruation hygiene visiting a public healthcare institute of Quetta, Pakistan
Source: BMC Womens Health. 2020 Jan 6;20:4. doi: 10.1186/s12905-019-0874-3 (PMC6945726; doi:10.1186/s12905-019-0874-3)
Supplement: Supplementary file 1 — Additional file 1. Questionnaire used for data collection (English version). [file 12905_2019_874_MOESM1_ESM.docx]

**Knowledge and practice of adolescent females about menstruation and menstruation hygiene visiting a public healthcare institute of Quetta, Pakistan**

| **Part I: Socio-Demographic Variables** |
| --- |

| Age(in years) |  |
| --- | --- |
| Education |  |
| Religion | 1. Muslim 2. Christian 3. Hindu 4. Others |
| What is your father’s educational level? | 1. Illiterate 2. Literate 3. Primary 4. Secondary 5. College |
| What is your mother’s educational level? | 1. Illiterate 2. Literate 3. Primary 4. Secondary 5. College |
| What is the occupation of your father? | 1. Businessman 2. Government employee 3. Non Government employee 4. Unemployed 5. Retired 6. Other -------- |
| What is the occupation of your mother? | 1. Housewife 2. Government employee 3. Non- Government employee 4. Unemploye 5. Retired 6. Other -------- |
| Age at the beginning of menstrual period (menarche) | 1. Before 11 years 2. 12-14 years 3. 15-16 years 4. Above 16 years |

| **Part II: Source of information regarding menstruation** |
| --- |

| What were the sources of knowledge towards menstruation before beginning of the menstrual period? | 1. Mother 2. Elder sister 3. Aunt 4. Friend 5. Media 6. Others------- |
| --- | --- |
| What was the information given about menstruation before beginning of menstrual period? | 1. Physical changes 2. Social Religious restrictions 3. Bathing processes 4. Use of the material for absorption 5. The blood is dirty 6. The blood comes from the vagina 7. None |
| Before the onset of menstruation, have you had any class session related to in your school? | 1. Yes 2. No |

| **Part III: Knowledge of respondents regarding menstruation** |
| --- |

| What is menstruation? | 1. Physiological Process 2. Disease 3. Curse of God 4. Others 5. Don’t know |
| --- | --- |
| What is the cause of menstruation? | 1. Hormones 2. Curse of God 3. Disease 4. Others 5. Don’t know |
| From which organ does the menstrual blood comes from? | 1. Uterus 2. Vagina 3. Bladder 4. Abdomen 5. Others 6. Don’t know |
| At what age do you think girls usually get their first period? | -------------- years |
| Do you know how to use a sanitary pad? | 1. Yes 2. No |
| Do you know that girls should take more nutritious diet during their periods? | 1. Yes 2. No 3. Do not know |
| What is the average duration of your menstruation flow? | ………… days |
| Do you think the menstrual blood is unhygienic? | 1. Yes 2. No |

| **Part IV: Problems faced by respondents during menstruation** |
| --- |

| Do you have any problems associated with menstruation? | 1. Headache 2. Vomiting 3. Weakness 4. Anorexia 5. Abdominal pain 6. Back pain 7. Others |
| --- | --- |
| Have you missed school because of menarche? | 1. Yes 2. No |
| Do you ever miss other activities (sports, games, social gatherings, etc.) during menstruation? | 1. Not at all 2. Sometimes 3. Rarely 4. Always |
| Do you avoid certain foods during menstruation? | 1. Yes 2. No |
| Do you think there is foul smell during menstruation? | 1. Yes 2. No |

| **Part V: Reaction of the respondents towards menstruation** |
| --- |

| What was the reaction to your first menstruation? | 1. Happy 2. Scared 3. Discomfort 4. Emotional disturbance 5. Others |
| --- | --- |
| What are your eating habits during menstruation? | 1. Eat less 2. Eat more 3. Eat same amount of food |
| Do you use absorbent material during period? | 1. Yes 2. No |
| What absorbent material do you use during menstruation? (You may choose more than one option) | 1. Commercially made sanitary napkin/pad 2. Homemade pads 3. Cotton wool 4. Nothing 5. Other |
| Do you take any medications for problems associated with menstruation | 1. Yes 2. No |
| What other remedies do you use to ease the discomfort of menstruation? | 1. Rest 2. Oil massage 3. Turmeric milk 4. Hot bottle packs 5. Others |
| When do you prefer to take bath during menstrual period? | 1. Daily 2. First day 3. Second day 4. Not during the period 5. Others |
| Do you clean your genitalia during menstruation? | 1. Yes 2. No |
| If yes, mostly with what? | 1. Water and soap 2. Only with water 3. Tissue paper 4. Towel 5. Others |

| **Part VI: Handling of the used material by the study respondents** |
| --- |

| How do you handle the used material? | 1. Discard it 2. Wash and discard it 3. Wash and reuse |
| --- | --- |
| Where do you dispose off your used materials? | 1. Dustbin 2. Drains 3. Toilets 4. Burn them 5. Others |
| How many times a day do you change the absorbent cloth/pad | 1. Once 2. Twice 3. Thrice 4. More than 3 times |
