# Supplementary material for: Knowledge and practice of adolescent females about menstruation and menstruation hygiene visiting a public healthcare institute of Quetta, Pakistan
Source: BMC Womens Health. 2020 Jan 6;20:4. doi: 10.1186/s12905-019-0874-3 (PMC6945726; doi:10.1186/s12905-019-0874-3)
Supplement: Supplementary file 2 — Additional file 2. Questionnaire used for data collection [Urdu (National language of Pakistan) version]. [file 12905_2019_874_MOESM2_ESM.docx]

# ‘حیض اور حیض حفظان صحت کے بارے میں نو عمر خواتین کا علم اور عمل کوئٹہ، پاکستان کے ایک صحت عامہ کے انسٹی ٹیوٹ کا دورہ کرتے ہوے’

| پارٹ ۱: سماجی و آبا دیاتی متغیر ات | |
| --- | --- |
|  | **عمر (سالوں میں)** |
|  | **تعلیم** |
| **۱۔ مسلمان**  **۲۔ عیسائی**  **۳۔ ہندو**  **۴۔ دوسرے** | **مزہب** |
| **۱۔ ان پڑھ**  **۲۔ پڑھے لکھے**  **۳۔ پرائمری**  **۴۔ سیکنڈری**  **۵۔ کالج** | **آپ کے والد کی تعلیمی سطح کیا ہے؟** |
| **۱۔ ان پڑھ**  **۲۔ پڑھے لکھے**  **۳۔ پرائمری**  **۴۔ سیکنڈری**  **۵۔ کالج** | **آپ کی والدہ کی تعلیمی سطح کیا ہے؟** |
| **۱۔ کاروباری**  **۲۔ سرکاری ملازم**  **۳۔ غیرسرکاری ملازم**  **۴۔ بےروزگار**  **۵۔ ریٹائر**  **۶۔ دوسرے** | **آپ کے والد کا پیشہ کیا ہے؟** |
| **۱۔ گھریلو خاتون**  **۲۔ سرکاری ملازمہ**  **۳۔ غیرسرکاری ملازمہ**  **۴۔ بےروزگار**  **۵۔ ریٹائر**  **۶۔ دوسرے** | **آپ کی والدہ کا پیشہ کیا ہے؟** |
| ۱**۔ ۱۱ سال سے پہلے**  **۲۔ ۱۲۔۱۴ سال**  **۳۔ ۱۵۔۱۶ سال**  **۴۔ ۱۶ سال سے زیادہ** | **ماہواری کے آغاز میں عمر ( بلوغت)؟** |

| **پارٹ ۲: حیض سے متعلق معلومات کا ماخز** | |
| --- | --- |
| **۱۔ ماں**  **۲۔ بڑی بہن**  **۳۔ خالہ**  **۴۔ دوست**  **۵۔ میڈیا**  **۶۔ دوسرے** | **ماہواری کے آغاز سے پہلے ماہواری کی طرف علم کے کیا زرائع تھے؟** |
| **۱۔ جسمانی تبد یلیا ں**  **۲۔ سماجی و مزہبی پابند یا ں**  **۳۔ غسل کے عمل**  **۴۔ جزب کے لیئے مواد کا استعمال**  **۵۔ خون گندہ ہے**  **۶۔ خون اندام نہانی سے آتا ہے**  **۷۔ کوئی نہیں** | **ماہواری کے آغاز سے پہلے ماہواری کے بارے میں کیا معلومات دی گئیں؟** |
| **۱۔ جی ہاں**  **۲۔ جی نہیں** | **ماہواری کے آغاز سے پہلے کیا آپ کے سکول میں اس سے متعلق کوئی سیشن ہوا؟** |

| **پارٹ ۳: حیض سے متعلق جواب دہندگان کا علم** | |
| --- | --- |
| **۱۔ جسمانی عمل**  **۲۔ بیماری**  **۳۔ خدا کی لعنت**  **۴۔ دوسرے**  **۵۔ پتہ نہیں** | **حیض کیا ہے؟** |
| **۱۔ ہارمونز**  **۲۔ خدا کی لعنت**  **۳۔ بیماری**  **۴۔ دوسرے**  **۵۔ پتہ نہیں** | **حیض کی وجہ کیا ہے؟** |
| **۱۔ بچہ دانی سے**  **۲۔ اندام نہانی سے**  **۳۔ مثانےسے**  **۴۔ پیٹ سے**  **۵۔ دوسرے**  **۶۔ پتہ نہیں** | **ماہواری خون کس عضو سے آتا ہے؟** |
| **۔۔۔۔۔۔۔۔۔۔۔۔۔۔۔ سال** | **آپ کے خیال میں لڈکیوں کو پہلا حیض کس عمر میں آتا ہے؟** |
| **۱۔ جی ہاں**  **۲۔ جی نہیں** | **کیا آپ جانتے ہیں کہ سینیٹری پیڈ کا استعمال کیسے کیا جاتا ہے؟** |
| **۱۔ جی ہاں**  **۲۔ جی نہیں**  **۳۔ پتہ نہیں** | **کیا آپ جانتے ہیں کہ لڑکیوں کو اپنے حیض کے دوران غذائیت سے بھرپور غذا لینی چاہئے؟** |
| **۔۔۔۔۔۔۔۔۔۔۔۔۔۔۔۔۔۔ دن** | **آپ کے حیض کے بہاؤ کی اوسط مدت کتنی ہے؟** |
| **۱۔ جی ہاں**  **۲۔ جی نہیں** | **کیا آپ کو لگتا ہے کہ حیض کا خون غیر صحت بخش ہے؟** |

| پارٹ ۴: ماہواری کے دوران جواب دہندگان کو جن دشواریوں کا سامنا کرنا پڑتا ہے | |
| --- | --- |
| **۱۔ سر درد**  **۲۔ الٹی**  **۳۔ کمزوری**  **۴۔ بھوک کی کمی**  **۵۔ پیٹ میں درد**  **۶۔ کمر میں درد**  **۷۔ دوسرے** | **کیا آپ کو ماہواری سے وابستہ کوئی پریشانی ہے؟** |
| **۱۔ جی ہاں**  **۲۔ جی نہیں** | **کیا آپ نے کبھی حیض کی وجہ سے سکول سے چھوٹی کی؟** |
| ***۱۔ بالکل نہیں***  ***۲۔ کبھی کبھی***  ***۳۔ شاذ و نادر ہی***  ***۴۔ ہمیشہ*** | **کیا آپ حیض کے دوران کبھی بھی دیگر سرگرمیوں (کھیل کود ، سماجی اجتماعات وغیرہ) سے محروم رہیں؟** |
| **۱۔ جی ہاں**  **۲۔ جی نہیں** | **کیا آپ حیض کے دوران کچھ کھانوں سے پرہیز کرتی ہیں؟** |
| **۱۔ جی ہاں**  **۲۔ جی نہیں** | **کیا آپ کو لگتا ہے کہ حیض کے دوران بدبو آ رہی ہے؟** |

| پارٹ ۵: ماہواری کے بارے میں جواب دہندگان کا رد عمل | |
| --- | --- |
| **۱۔ خوش**  **۲۔ ڈری ہوئی**  **۳۔ تکلیف**  **۴۔ جذباتی پریشانی**  **۵۔ دوسرے** | **آپ کے پہلے حیض پر رد عمل کیا تھا؟** |
| **۱۔ *کم کھاتی ہیں***  ***۲۔ زیادہ کھاتی ہیں***  ***۳۔ اسی مقدار میں کھاتی ہیں*** | **حیض کے دوران آپ کو کھانے کی کیا عادات ہیں؟** |
| **۱۔ جی ہاں**  **۲۔ جی نہیں** | **کیا آپ حیض کے دوران جاذب مواد استعمال کرتی ہیں؟** |
| **۱*۔ دوکانوں پر ملنے والے سینٹری نیپکن / پیڈ***  ***۲۔ گھریلو پیڈ***  ***۳۔ کپاس کی اون***  ***۴۔ کچھ نہیں***  ***۵۔ دیگر*** | **حیض کے دوران آپ کون سا جاذب مواد استعمال کرتی ہیں؟ (آپ ایک سے زیادہ اختیارات منتخب کرسکتی ہیں)** |
| **۱۔ جی ہاں**  ۲۔ جی نہیں | **کیا آپ حیض سے وابستہ مسائل کے لئیے کوئی دوا لیتی ہیں؟** |
| ۱۔ آرام  ۲۔ تیل مساج  ۳۔ ہلدی کا دودھ۔  ۴۔ گرم بوتل کے پیک  ۵۔ دوسرے | **ماہواری کی تکلیف کو کم کرنے کے لئے آپ کون سے دوسرے علاج استعمال کرتی ہیں؟** |
| *۱۔ روزانہ*  **۲۔ پہلے دن**  **۳۔ دوسرے دن**  **۴۔ حیض کے دوران نہیں**  **۵۔ دوسرے** | **آپ ماہواری کے دوران کب غسل کرنا پسند کرتی ہیں؟** |
| **۱۔ جی ہاں**  **۲۔ جی نہیں** | **کیا آپ حیض کے دوران اپنے تناسل کو صاف کرتے ہیں؟** |
| ***۱۔ پانی اور صابن***  ***۲۔ صرف پانی کے ساتھ***  ***۳۔ ٹشو پیپر***  ***۴۔ تولیہ***  ***۵۔ دوسرے*** | **اگر ہاں ، تو زیادہ تر کس کے ساتھ؟** |

| پارٹ ۶: جواب دہندگان کا استعمال شدہ مواد کو ہینڈل کرنا | |
| --- | --- |
| **۱۔ اسے خارج کردیتی ہیں**  **۲۔ اسے دھو کر چھوڑ دیتی ہیں**  **۳۔ دھو کر دوبارہ استعمال کرتی ہیں** | **آپ استعمال شدہ مواد کو کس طرح سنبھالتی ہیں؟** |
| ***۱۔ ڈسٹ بِن***  ***۲۔ نالے***  **۳۔ ٹوالیٹ**  **۴۔ ان کو جلا دیتی ہیں**  **۵۔ دوسرے** | **آپ اپنے استعمال شدہ سامان کو کہاں نپٹاتی ہیں؟** |
| ***۱۔ ایک بار***  ***۲۔ دو بار***  ***۳۔ تین بار***  ***۴۔ 3 بار سے زیادہ*** | **آپ دن میں کتنی بار جاذب کپڑا / پیڈ تبدیل کرتی ہیں۔** |
